# Epidemiological Survey of Enterovirus Infections in Taiwan From 2011 to 2020: Retrospective Study

**DOI:** 10.2196/59449

**Published:** 2024-09-05

**Authors:** Fang-Chen Liu, Bao-Chung Chen, Yao-Ching Huang, Shi-Hao Huang, Ren Jei Chung, Pi-Ching Yu, Chia-Peng Yu

**Affiliations:** 1 Department of Internal Medicine, Tri-Service General Hospital, National Defense Medical Center, Taipei City, Taiwan; 2 Department of Chemical Engineering and Biotechnology, National Taipei University of Technology (Taipei Tech), Taipei, Taiwan; 3 Graduate Institute of Medicine, National Defense Medical Center, Taipei, Taiwan; 4 Cardiovascular Intensive Care Unit, Department of Critical Care Medicine, Far-Eastern Memorial Hospital, New Taipei City, Taiwan; 5 School of Public Health, National Defense Medical Center, Taipei, Taiwan

**Keywords:** epidemiology, enterovirus, domestic, cluster, sporadic, retrospective, Taiwan

## Abstract

**Background:**

Young children are susceptible to enterovirus (EV) infections, which cause significant morbidity in this age group.

**Objective:**

This study investigated the characteristics of virus strains and the epidemiology of EVs circulating among young children in Taiwan from 2011 to 2020.

**Methods:**

Children diagnosed with EV infections from 2011 to 2020 were identified from the routine national health insurance data monitoring disease system, real-time outbreak and disease surveillance system, national laboratory surveillance system, and Statistics of Communicable Diseases and Surveillance Report, a data set (secondary data) of the Taiwan Centers for Disease and Control. Four primary outcomes were identified: epidemic features, characteristics of sporadic and cluster cases of EV infections, and main cluster institutions.

**Results:**

From 2011 to 2020, between 10 and 7600 person-times visited the hospitals for EV infections on an outpatient basis daily. Based on 2011 to 2020 emergency department EV infection surveillance data, the permillage of EV visits throughout the year ranged from 0.07‰ and 25.45‰. After typing by immunofluorescence assays, the dominant type was coxsackie A virus (CVA; 8844/12,829, 68.9%), with most constituting types CVA10 (n=2972), CVA2 (n=1404), CVA6 (n=1308), CVA4 (n=1243), CVA16 (n=875), and CVA5 (n=680); coxsackie B virus CVB (n=819); echovirus (n=508); EV-A71 (n=1694); and EV-D68 (n=10). There were statistically significant differences (*P*<.001) in case numbers of EV infections among EV strains from 2011 to 2020. Cases in 2012 had 15.088 times the odds of being EV-A71, cases in 2014 had 2.103 times the odds of being CVA, cases in 2015 had 1.569 times the odds of being echovirus, and cases in 2018 had 2.274 times the odds of being CVB as cases in other years. From 2011 to 2020, in an epidemic analysis of EV clusters, 57 EV clusters were reported. Clusters that tested positive included 53 (53/57, 93%) CVA cases (the major causes were CVA6, n=32, and CVA10, n=8). Populous institutions had the highest proportion (7 of 10) of EV clusters.

**Conclusion:**

This study is the first report of sporadic and cluster cases of EV infections from surveillance data (Taiwan Centers for Disease and Control, 2011‐2020). This information will be useful for policy makers and clinical experts to direct prevention and control activities to EV infections that cause the most severe illness and greatest burden to the Taiwanese.

## Introduction

Enteroviruses (EVs) are among the most common viruses infecting humans and are responsible for an estimated 10 to 15 million cases of symptomatic infections annually in the United States [1]. Furthermore, in the United States, children <1 year old may account for ~40% of EV infections in patients with a known age [2]. Verboon-Maciolek et al [3] estimated an incidence of EV infections in the neonatal period of 26 cases per 100,000 live births in The Netherlands. Nonpolio EVs (NPEVs) show seasonal patterns of incidence. In temperate areas, EVs are characteristically found in summer and early autumn, although outbreaks may continue into winter. In tropical climates, circulation is year-round or associated with the rainy season. In Europe, 50% to 65% of infant infections occur from April to October. There are frequent fluctuations in predominant EV serotypes, with some serotypes showing periods of relative quiescence followed by extensive outbreaks. Variation by location is an important characteristic of EV epidemiology [4-9]. Patients with uncomplicated EV illness bring significant economic and medical impacts on society with at least 1 to 4 days of missed school or lost work, direct medical costs of US $69 to US $771 per case, and indirect costs of US $63 to US $422 per case, mainly attributable to parental missed work [10,11].

EV spreads from person to person by fecal-oral and respiratory routes, but indirect transmission may also occur via different routes, including contaminated water, food, and fomites [12]. Neonatal EV infections can be acquired antenatally (transplacentally or via an ascending route), intrapartum (usually with clinical presentation from 2 to 7 days of life), and post natally (usually by family members but sometimes from health care workers) [13,14]. Inadequate handwashing can contribute to the spread of EV infections in the neonatal intensive care unit; however, vertically acquired infections are usually more severe than horizontally acquired ones [15]. A recent study on neonates and young infants in Japan has reported EV-positive stool samples in 91% of siblings and 42% of parents. Most (45% of siblings and 85% of parents) were asymptomatic [16].

EVs are among the most prevalent viruses infecting humans worldwide [17]. There are 106 types known to infect humans, of which dozens are frequently detected globally [18-21]. Most of these types have been defined before sequencing techniques are available on a large scale, and typing is performed based on the biological properties of these viruses in cell culture or mouse models. EVs are a genus of small, single-stranded, positive-sense RNA viruses of the family *Picornaviridae* [22]. EV types identified during that time are still called coxsackie A virus (CVA), coxsackie B virus (CVB), echoviruses (E), numbered EV (EV-A, -B, -C, and -D), and polioviruses. The newer types have been defined by analysis of their viral protein 1 region sequence, and their names consist of “enterovirus,” their species letter, and a consecutive number, starting with EV-D68 [23].

EVs cause a wide spectrum of diseases, including encephalitis; meningitis; myocarditis; hand, foot, and mouth disease (HFMD); conjunctivitis; respiratory diseases; and gastrointestinal diseases, but most infections remain asymptomatic [24-30]. Many prevalent studies have been conducted to describe the EV prevalence and the distribution of distinct EV types in cohorts of patients with specific symptoms, such as gastroenteritis, influenza-like illness, and meningitis, and asymptomatic cohorts. Surveillance studies have reported the EV prevalence and EV types detected in larger populations with less well-defined symptoms, such as all individuals tested for EVs at a national level [19,21].

Taiwan has become a developed country with a per capita gross domestic product of US $35,244 [31]. There are sporadic and cluster cases of EV infections in Taiwan, and children have the highest rate of getting EV. The course of the disease often causes severe illness and medical burden [32,33]. Therefore, this study aimed to use the TCDC statistics of communicable diseases and surveillance report to explore the number of EV outpatient and emergency department visits for sporadic and cluster cases from 2011 to 2020. The epidemiological characteristics, differences, and trends of EVs and clustered virus strains are the main clustering areas of EVs.

## Methods

### Ethical Considerations

This study used information freely available in the public domain and analyzed open datasets where data have been properly anonymized. Ethics approval for human participant research is, therefore, exempt. The original informed consent allows for secondary analysis without additional consent. This study conforms with the public use of government reports [34,35].

### Definition of Sporadic Reported and Confirmed Cases

Sporadic and cluster cases of EV infections are nationally notifiable in Taiwan [36]. To reinforce surveillance efficiency, the TCDC established a sentinel surveillance system across Taiwan. According to the Communicable Disease Control Act of Taiwan, all sporadic cases of suspected EV infections must be reported, and a specimen must be collected and sent to the TCDC through the “national laboratory surveillance system.” Suspected cases should be patients with HFMD or herpangina, and their specimens should be collected within 3 days after the onset of illness. Sources of specimens (throat swabs, rectal swabs, stools, sera, or cerebrospinal fluid) at the contracted laboratories were mainly from outpatients, emergency, and inpatient patients at medical centers within the areas covered by the laboratories and from 165 specimen collection stations nationwide. Sporadic cases were defined as persons hospitalized (or outpatient and emergency clinic) for community-onset, laboratory-confirmed EV infections by immunofluorescence assays (IFAs) or reverse transcription–polymerase chain reaction. At first, viruses were isolated following the standard protocols for the EV surveillance system conducted by the TCDC, as described previously [37]. EV isolates were identified by IFAs using commercial antibodies against EV (Light Diagnostics; Millipore) according to the manufacturer’s protocol [38]. All IFA-untypeable EVs were tested by traditional reverse transcription–polymerase chain reaction and sequencing [39]. The QIAamp Viral RNA Mini Kit (Qiagen) was used for RNA extraction according to the manufacturer’s instructions. The EV Consensus-Degenerate Hybrid Oligonucleotide Primers (CODEHOP) method was used to identify untypeable EVs [40].

This study analyzed the “real-time outbreak and disease surveillance system” data of the TCDC. The 180 responsible hospitals in Taiwan were also used to transmit information about EV emergency medical treatment through the internet to the TCDC. The TCDC collects and analyzes the “real-time outbreak and disease surveillance system” secondary data weekly, makes statistical charts, and publishes them on the government’s public website for the academic community (including this study). Furthermore, to strengthen Taiwan’s surveillance capacity for specific diseases, the TCDC and the National Health Insurance Administration of Taiwan embarked on horizontal cooperation, under which the Taiwan’s Bureau of National Health Insurance compiles the outpatient data uploaded by hospitals and clinics through the National Health Insurance IC cards. The TCDC conducts daily routine surveillance of specific diseases based on the comprehensive and highly representative secondary statistical data compiled by the Taiwan’s Bureau of National Health Insurance to assess the magnitude of an epidemic. This was called the “routine national health insurance data monitoring disease system.” The TCDC releases data to the government’s public website for analysis by the academic community (including this study).

### Definition of Reported and Confirmed Cases of Cluster Events

Patients and their contacts among high-risk groups for EV infections with severe complications are involved in a suspected cluster of EV infections in places such as nurseries and neonatal wards in hospitals, baby care centers, and homes of puerperal care, except schools [36]. Officers at the local Department of Health and Regional Center of the TCDC are responsible for collecting specimens from selected patients and submit to the Center for Research, Diagnostics, and Vaccine Development of the TCDC. Cluster cases were defined as described previously by the diagnostic methods of sporadic cases. Patients and their contacts who are among high-risk groups for EV infection with severe complications and are involved in a suspected cluster of EV infection in places such as nurseries and neonatal wards in hospitals, babycare centers, and homes of puerperal care. The TCDC publishes secondary data on the government’s public website for the academic community (including this study).

### Data Analysis

This was a retrospective historical study of all sporadic and cluster cases of EV infections since 2011. The number of people diagnosed as having EV infections from 2011 to 2020 was confirmed, and the distribution of their epidemiological characteristics, differences, and results was examined. Viral strains of sporadic and cluster cases and the related results in the analysis of cases of EV infections from 2011 to 2020 were emphasized. Descriptive data were shown as the mean and summary, where appropriate. Categorical variables were compared using the *χ*^2^ test. All statistical analyses were performed using SPSS (version 21; IBM Corp). All statistical tests were 2-sided with α=.05. *P* values <.05 were considered statistically significant.

## Results

**Figure 1.**
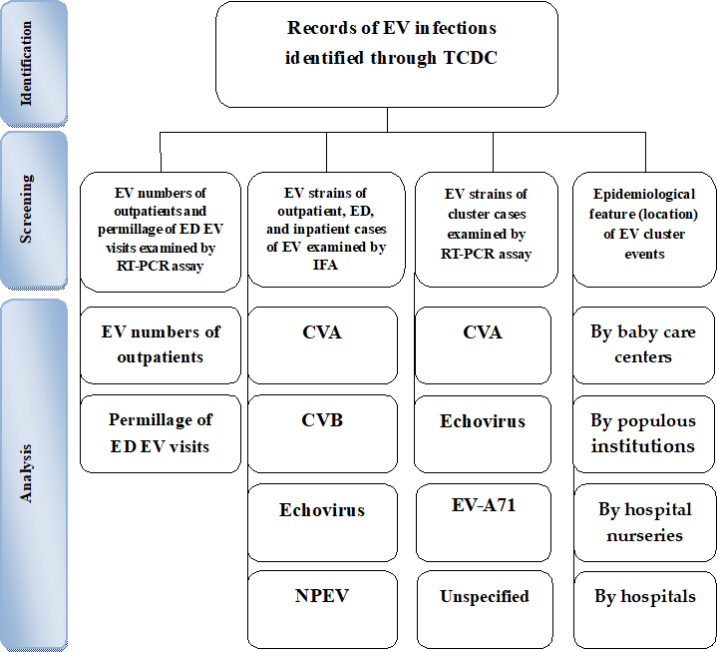
Flowchart of the study sample selection from the TCDC database in Taiwan from 2011 to 2020. CVA: coxsackie A virus; CVB: coxsackie B virus; ED: emergency department; EV: enterovirus; IFA: immunofluorescence assay; NPEV: nonpolio enterovirus; RT-PCR: reverse transcription–polymerase chain reaction; TCDC: Taiwan Centers for Disease and Control.

The research flowchart of this study is shown in Figure 1. From 2011 to 2020, Taiwan saw a spike in EV infection prevalence. Based on disease surveillance using national health insurance data, the number of person-times who visited the hospitals for EV infections on an outpatient basis daily ranged from 10 to 300, 100 to 5100, 200 to 7600, 89 to 7730, 99 to 4925, 155 to 6253, 48 to 3888, 30 to 2670, 60 to 4783, and 27 to 1575, respectively (Table 1).

Furthermore, 239 EV strains were isolated in 2020. After typing by IFA, the dominant type was CVA (n=178 strains, 74.5%), with most constituting type CVA6 (n=78 strains), CVA5 (n=51 strains), and CVA2 (n=25 strains); CVB (n=3 strains, 1.3%); EV-A71 (n=4 strains, 1.7%); and NPEV (n=54 isolates, 22.6%; the major causes were rhinovirus). In sum, the top 5 EVs isolated in 2020 were CVA6 (32.6%), CVA5 (21.3%), CVA2 (10.5%), CVA16 (5.4%), and CVA4 (4.6%; Table 2).

There were statistically significant differences (*P*<.001) in case numbers of EV infections among EV strains from 2011 to 2020 (Table 3). Cases in 2012 had 15.088 times the odds of being EV-A71, cases in 2014 had 2.103 times the odds of being CVA, cases in 2015 had 1.569 times the odds of being echovirus, and cases in 2018 had 2.274 times the odds of being CVB as cases in other years.

**Table 1.** Daily outpatient visits for enteroviruses and the annual permillage of emergency department visits in Taiwan from 2011 to 2020.

Variables	Year									
	2011(n=24,200)	2012 (n=13,502)	2013 (n=11,464)	2014 (n=11,387)	2015 (n=10,563)	2016(n=12,671)	2017 (n=11,665)	2018 (n=10,421)	2019 (n=10,026)	2020 (n=11,199)
Daily outpatient visits (person)	10‐3300	100‐5100	200‐7600	89‐7730	99‐4925	155‐6253	48‐3888	30‐2670	60‐4783	27‐1575
The annual permillage of emergency department visits (‰)	0.07‐9.21	0.81‐15.37	1.31‐25.45	0.75‐19.67	0.56‐11.60	0.64‐16.28	0.70‐10.67	0.52‐6.67	1.00‐12.48	0.08‐4.17

**Table 2.** Distribution characteristics of enterovirus strains in outpatient, emergency, and inpatient patients in Taiwan from 2011 to 2020.

Variables		Distribution characteristics from years 2011-2020									
		2011 (n=2171)^a^	2012 (n=1742)	2013 (n=1005)	2014 (n=1170)	2015 (n=1046)	2016 (n=1899)	2017 (n=802)	2018 (n=1447)	2019 (n=1308)	2020 (n=239)
**CVA^b^** **(%)**		n=1658	n=605	n=761	n=953	n=704	n=1519	n=575	n=960	n=931	n=178
	CVA10	51.8	1.6	6.5	32.1	3.8	29.0	3.1	25.8	22.1	—^c^
	CVA 16	0.6	0.6	2.4	7.4	29.1	5.2	2.9	16.3	5.0	5.4
	CVA 2	4.3	22.8	4.7	18.6	0.9	15.4	18.7	0.6	12.5	10.5
	CVA 4	9.9	5.7	—	—	15.8	7.2	21.1	18.4	13.8	4.6
	CVA 5	3.4	0.1	6.2	9.7	—	17.3	—	0.4	3.3	21.3
	CVA 6	0.9	3.8	52.5	0.6	16.0	4.8	20.8	1.8	12.1	32.6
	CVA 9	5.1	—	—	1.1	1.6	1.1	5.0	3.1	1.8	—
	CVA 8	—	—	0.1	—	—	—	—	—	0.1	—
	CVA 21	—	—	0.5	0.3	—	—	—	—	0.3	—
	CVA 24	—	—	0.2	—	—	—	—	—	—	—
	CVA 1	—	—	6.1	11.6	—	—	—	—	—	—
	Untype	0.4	0.1	0.4	—	—	—	—	—	—	—
**CVB^d^ (%)**		n=93	n=80	n=78	n=87	n=108	n=95	n=55	n*=*174	n=46	n=3
	CVB 1	0.2	—	0.1	1.8	0.5	—	0.7	0.9	—	0.4
	CVB 2	0.1	0.1	4.1	0.8	—	—	0.4	1.2	0.1	—
	CVB 4	0.3	1.4	4.4	0.3	—	1.6	1.5	1.0	2.1	0.4
	CVB 5	3.6	0.2	0.1	4.5	8.7	0.6	2.6	8.9	1.1	—
	CVB 3	—	2.9	0.5	—	0.6	2.7	1.6	0.1	0.2	0.4
	untype	—	—	—	—	—	—	—	—	0.1	—
**E^e^ (%)**		n=13	n=46	n=12	n=18	n=61	n=35	n=39	n=231	n=53	n=0
	E 30	0.1	0.8	1.1	—	1.5	0.3	—	—	—	—
	E 4	0.1	1.1	0.2	0.6	0.8	0.2	0.2	0.1	0.2	—
	E 6	0.2	0.4	—	0.5	2.7	1.2	3.2	0.3	3.1	—
	E 9	0.2	0.3	—	0.3	0.7	—	—	—	0.3	—
	E 11	—	—	—	0.1	—	—	0.5	15.5	0.2	—
	E 5	—	—	—	—	—	—	0.7	—	—	—
	E 18	—	—	—	—	—	—	—	0.1	0.2	—
	E 25	—	—	—	—	—	—	—	—	0.1	—
EV^f^-A71, n (%)		349 (16.1)	923 (53.0)	21 (2.1)	1 (0.1)	17 (1.6)	90 (4.7)	16 (2.0)	46 (3.2)	227 (17.3)	4 (1.7)
EV-D68, n (%)		—	—	—	—	—	—	7 (0.9)	—	3 (0.2)	—
Unspecific, n (%)^g^		58 (3.0)	88 (5.0)	133 (13.2)	111 (9.0)	156 (15.0)	160 (8.4)	110 (13.7)	36 (2.5)	48 (4.0)	54 (22.6)
Sample number		24,200	13,502	11,464	11,387	10,563	12,671	11,665	10,421	10,026	11,199

a Denominator of permillages is 2171 (cases) for the year 2011. The remaining numbers in the denominator are the number of EV cases in each year.

b CVA: coxsackie A virus.

c Not applicable.

d CVB: coxsackie B virus.

e E: echovirus.

f EV: enterovirus.

g Unspecific: gene sequencing only shows the type of virus strain, but not the number, so it cannot be classified as above genotype.

**Table 3.** Association between year and virus strain from a survey of confirmed cases of EV infections between 2011 and 2020 in Taiwan.

Variable	Year										*P* value
	2011 (n=2171)	2012 (n=1742)	2013 (n=1005)	2014 (n=1170)	2015 (n=1046)	2016 (n=1899)	2017 (n=802)	2108 (n=1447)	2019 (n=1308)	2020 (n=239)	
CVA^a^ (%)	1658	605	761	953	704	1519	575	960	931	178	<.001
CVB^b^ (%)	93	80	78	87	108	95	55	174	46	3	<.001
E^c^ (%)	13	46	12	18	61	35	39	231	53	0	<.001
EV^d^-A71 (%)	349	923	21	1	17	90	16	46	227	4	<.001
EV-D68 (%)	—^e^	—	—	—	—	—	7	—	3	—	<.001
Unspecified (%)	58	88	133	111	156	160	110	36	48	54	<.001

a CVA: coxsackie A virus.

b CVB: coxsackie B virus.

c E: echovirus.

d EV: enterovirus.

e Not applicable.

In 2020, an epidemic analysis of EV clusters reported 11 EV clusters. Clusters that tested positive included 5 CVA6 infections and 1 CVA4 infection. The other events were negative or had no specimens taken (Table 4). Populous institutions had the highest number of EV clusters (Table 5).

**Table 4.** Characterization of clusters of enterovirus infection cases by viral genotype from 2011 to 2020 in Taiwan.

Variables			Year									
			2011	2012	2103	2014	2015	2016	2017	2018	2019	2020
EV^a^-reported cluster events, n			1	0	3	0	2	2	8	13	48	11
**EV-confirmed cluster events, n**			1	0	2	0	2	1	5	6	34	6
	**Virus genotype**											
		CVA2^b^	0	0	1	0	0	0	0	0	4	0
		CVA6	0	0	1	0	1	0	3	3	16	5
		CVA4+E4^c^+E18	0	0	0	0	1	0	0	0	0	0
		CVA10	0	0	0	0	0	1	1	2	4	0
		EV-A71	0	0	0	0	0	0	1	0	2	0
		CVA4	0	0	0	0	0	0	0	1	4	1
		CVA6+adenovirus	0	0	0	0	0	0	0	0	1	0
		CVA6+EV-A71	0	0	0	0	0	0	0	0	1	0
		CVA6+CVA2	0	0	0	0	0	0	0	0	1	0
		CVA5	0	0	0	0	0	0	0	0	1	0
		Unspecified	1	0	0	0	0	0	0	0	0	0

a EV: enterovirus.

b CVA: coxsackie A virus.

c E: echovirus.

**Table 5.** Characterization of clusters of enterovirus infection cases by location from 2011 to 2020 in Taiwan.

Variable		Year									
		2011 (n=1)	2012 (n=0)	2013 (n=3)	2014 (n=0)	2015 (n=2)	2016 (n=2)	2017 (n=8)	2018 (n=13)	2019 (n=48)	2020 (n=11)
**Major institutions** ^**a**^											
	Baby care centers	✓^b^				✓					
	Populous institutions			✓		✓	✓	✓	✓	✓	✓
	Hospital nurseries			✓							
	Hospitals							✓	✓		

a Suspected enterovirus clusters in schools do not require notification in this study.

b Only list major cluster institutions; did not included numbers.

## Discussion

As Taiwan is a densely populated island and a major air and sea port, the importation and dissemination of viral infections are facilitated [41]. Seasonal variation occurs with EV and other respiratory virus infections. EVs are suitable for survival and transmission in humid and hot environments. Taiwan is in the subtropical zone, and infection cases occur yearly. Therefore, EV infection is one of the endemic diseases in Taiwan. According to domestic monitoring data over the years, young children are a high-risk group for infections complicated by severe disease and death, and the fatality rate of severe disease is 1.3% to 33.3% [42]. Using the “routine national health insurance data monitoring disease system” [36] and “real-time outbreak and disease surveillance system” [36] from 2011 to 2020, this study found that in the survey period, between 20 and 7600 person-times visited the hospitals for EV infections on an outpatient basis daily, which was highest in 2013 (between 200 and 7600 person-times daily). Furthermore, based on the 2011 to 2020 emergency EV infection surveillance data, the permillage of EV visits throughout the year ranged from 0.07‰ to 25.45‰, which was highest in 2013 (between 1.31‰ and 25.45‰). The dominant type was CVA (931 strains or 71.2%), followed by CVB, EV-A71, echovirus, and EV-D68 by the “national laboratory surveillance system” [36]. CVA is the main pathogen of EV strains among cluster events in Taiwan. The annual epidemic situation in Taiwan is partly affected by changes in the type of virus strains or the genetic variation of the pathogen of EVs. The scale of infection and severity of the disease would also vary. Long-term community monitoring is required to detect early changes in EV strains [43]. Therefore, this study suggested that establishing long-term and stable community-based monitoring can provide information on the activity of EVs in different regions and seasons in Taiwan, conduct epidemic prevention preparations in advance, and help control EV epidemics.

In Taiwan, EV infections primarily affect young children [44]. The genus *enterovirus* of the family Picornaviridae includes polioviruses, CV, echoviruses, numbered EVs, and rhinoviruses [45]. For decades, most studies focused on poliovirus, but knowledge of NPEVs has increased considerably in recent years. Several NPEVs have emerged as serious public health concerns. HFMD, a frequently reported and concerning disease worldwide, is a severe burden on societies globally, especially in East and Southeast Asian countries [46]. Han et al [46] indicated that CVA16 is one of the most important causes of HFMD in China and a severe threat to human health, especially in children <5 years old. A previous study in Vietnam indicated that HFMD is a major public health concern in the Asia Pacific region. Most recent HFMD outbreaks have been caused by EV-A71, CVA16, CVA10, and CVA6 [47]. EV strains circulating in China or Vietnam were similar to those in this study. During the 10-year survey period, the main circulating virus strains in Taiwan included CVA2, CVA4, CVA5, CVA6, CVA10, CVA16, and EV-A71. In particular, CVA2, CVA6, and CVA16 circulate among susceptible hosts in Taiwan yearly, a major burden for medical clinical and epidemic prevention in the country. Moreover, a previous study indicated that EV-A71, CVA16, and CVA6 are generally the most common causative pathogens for HFMD, whereas EV-A71 is the most frequently identified serotype among severe and fatal cases [48-50]. Therefore, there are many EV serotypes, and virus strains circulating each year differ [51]. In addition to increasing the difficulty of estimating the epidemic cycle and the scale of the epidemic, in the year when the EV-A71 is prevalent, severe epidemics caused by other types may also occur (eg, EV-D68 in 2017 or E11 in 2018). Therefore, this study indicated that central and local health authorities should continue to implement various epidemic prevention preparations and strengthen the public’s ability of individuals to prevent epidemics to reduce the probability of EV transmission.

Previous studies indicated that EV-A71, CVA16, and CVA6 are common serotypes causing the HFMD outbreak in China [52]. A previous study also indicated that the phylogenetic tree of the partial viral protein 1 sequence showed that CVA6 isolates are divided into 4 clusters. The CVA6 cluster predominantly circulates in HFMD in China [53]. The EV-A71 subgenogroup C4 caused the largest outbreak of HFMD in Vietnam, China, Australia, and Italy [54-59]. These results were similar to this study. CVA6, CVA10, CVA4, and EV-A71 were the main EV strains in this study. In addition to monitoring and identifying pathogens, medical care is an important aspect of EV prevention and treatment. A few cases of EV infections may cause severe illness or death due to the rapid changes in the course of the disease. Therefore, this study suggested that in addition to continuing existing surveillance strategies and self-preventive care, future efforts should be to conduct empirical research on control strategies as soon as possible to provide effective control measures and reduce social costs.

Previous research indicated that empirical evidence on the role played by population density in spreading the coronavirus is based on cross-sectional data covering 172 countries (obtained from several sources, including the European Centre for Disease Prevention and Control, the World Bank, and the Center for Health Security). Results using extreme bounds analysis techniques and variable addition tests showed that population density significantly positively affects the number of cases [60]. Moreover, a previous study indicated a high frequency of EV serotype circulating in a densely populated area of India [61]. Cluster events are common because of the characteristics of EV infections. Considering the burden load of laboratory manpower and epidemic prevention, major cluster events (occurring in hospital baby rooms, neonatal wards, childcare centers, postpartum nursing homes, and other places) must be notified and inspected in Taiwan. This study also indicated that populous institutions are Taiwan’s main infectious areas of cluster events. Therefore, monitoring this disease and its epidemiology in the densely populated part of Taiwan is important for detecting EVs of emerging epidemics. This study suggested that to clarify the possible source of infection, it is necessary to conduct epidemic investigations on reported cluster cases, including the onset process, medical treatment, caregivers and coresidents, and activities during the incubation period. To test samples immediately after the notification, clinicians will diagnose the clinical symptoms and test results of the patients and follow up on epidemiological characteristics to facilitate the diagnosis and judgment of cases and subsequent epidemic control [62].

This study has 3 drawbacks. First, the statistics about infectious diseases disclosed by the TCDC on the internet only provide basic epidemiological data about EV infection patients, with no clinical data. Therefore, this study could not compare differences or trends in patient clinical symptoms. Second, the information disclosed on the platform also does not contain information about the genetic information of EVs. Neither the characteristics of the antigenic structure of EV prevalent in Taiwan nor the genetic relationship when comparing EV strains in this country to other countries remains unknown. Third, when a suspected EV cluster event occurs in schools and kindergartens, it is unnecessary to report and collect data in accordance with operational instructions for “real-time outbreak and disease surveillance system” and “national laboratory surveillance system” but should still follow the notification of mild cases of EV and cluster event management announced by the local government.

In conclusion, this study revealed the characteristics of virus strains and trends of sporadic and cluster cases of EV infections from 2011 to 2020. Over the past 10 years, Taiwanese children have continued to experience EV infections. The authors identified substantial patients with EV infection burden in children in Taiwan. The rate of CVA infection in children is still high, and acute and severe cases caused by EV-A71 and EV-D68 in recent years have become the focus of clinicians and public health. EV clustering caused by CVA6 requires continuous attention and long-term tracking of its trend changes. This study confirms that EV clusters occur most frequently in populous institutions. Individuals should reduce the frequency of entering densely populated institutions to prevent EV infections in high-risk populations, including children. The government is taking proactive measures against EV infections. This information will be useful for policy makers and clinical experts in directing prevention and control activities for patients with EV infections that burden the Taiwanese. These data will inform future surveillance and research efforts in Taiwan.

## Abbreviations

CODEHOP: Consensus-Degenerate Hybrid Oligonucleotide Primers
CVA: coxsackie A virus
CVB: coxsackie B virus
EV: enterovirus
HFMD: hand, foot, and mouth disease
IFA: immunofluorescence assay
NPEV: nonpolio enterovirus
TCDC: Taiwan Centers for Disease and Control
